# Nursing Students’ Experiences With the Flipped Classroom Approach in a Human Anatomy Course: A Qualitative Study

**DOI:** 10.1177/23779608261433108

**Published:** 2026-05-15

**Authors:** Vistolina Nuuyoma, Maria Shishiveni

**Affiliations:** 1 School of Nursing and Public Health, 99404 University of Namibia, Windhoek, Khomas Region, Namibia

**Keywords:** Active learning, bioscience teaching, constructivism, flipped classroom, human anatomy, inverted classroom

## Abstract

**Introduction:**

The flipped classroom is a blended teaching approach in which students receive content prior to attending face-to-face lessons. It typically comprises three phases: pre-class, in-class and post-class. Previous research has predominantly focused on the flipped classroom in general higher education, nursing education and anatomy education for medical students; however, its specific use in anatomy education among nursing students remains understudied.

**Objective:**

This study aimed to explore and describe nursing students’ experiences with a flipped classroom in a human anatomy course.

**Methods:**

The flipped classroom approach was used once a week for a second-semester human anatomy class for first-year undergraduate nursing students, while the other days adhered to the traditional approach. Subsequently, an exploratory, descriptive qualitative study was conducted, with constructivism as its theoretical foundation. A sample of 18 nursing students was selected through convenience sampling to participate in the study. Data were collected through individual interviews, using an interview guide. The data were transcribed verbatim and then manually thematically analysed, following six stages of the iterative process of analysing interview data.

**Results:**

The three themes identified are: the flipped classroom promotes active learning, the flipped classroom is not the preferred method for learning human anatomy, and improving the flipped classroom approach.

**Conclusion:**

Findings indicate both positive and negative experiences with the flipped classroom in human anatomy. These may help develop a step-by-step guide to teaching human anatomy using the flipped classroom approach. Future researchers might explore how the flipped classroom can be utilised to enhance the application of human anatomy in clinical practice among nursing students.

## Introduction

The flipped classroom has become a practical and innovative teaching approach in higher education and has gained popularity globally to foster active learning (Andargeery et al., 2024; Baig & Yadegaridehkordi, 2023). It is a blended teaching approach that presents students with content prior to attending face-to-face lessons (Wilson & Hobbs, 2023). Therefore, learning aids such as video-recorded lectures enable students to review the instructional content before attending class (Mistry, 2025). The prior learning content is structurally aligned with learning activities to be completed with instructors and peers on campus (Poudel & Sharma, 2022). According to Youhasan et al. (2021), the flipped classroom commonly involves three key phases: pre-class, in-class and post-class, with activities tailored for each phase. In the pre-class phase, students are expected to engage with the instructor-provided learning materials to gain sufficient exposure for the in-class phase. During the in-class phase, students undertake a series of learning activities such as discussions, debates, case studies, problem solving and group presentations with minimal support from the instructor(McLean & Attardi, 2023; Youhasan et al., 2021). The in-class time is utilised to the fullest for active learning activities. Then, in the post-class phase, students are assigned various assignment tasks or quizzes as enrichment activities to reinforce the knowledge they acquired during the earlier phases (Al-Samarraie et al., 2020). The flipped classroom is characterised as a constructivist approach to learning. Promoting social interactions and a collaborative learning approach, with students as active creators of their learning experiences, building on prior learning (Erbil, 2020).

### Review of Literature

The flipped teaching method has been used throughout history, although it has not been given a specific name. With the advancement of computers in the 1980s, the development of the internet in the 1990s, and the introduction of various online learning platforms in the 2000s, these eras facilitated the adoption of the flipped classroom (Gopalan et al., 2022). The flipped classroom approach gained prominence during the COVID-19 pandemic, particularly for facilitating the transition from face-to-face to remote teaching and learning (Beason-Abmayr et al., 2021). The flipped classroom has gained global adoption and is used in many different fields of study (Zhang et al., 2024). However, despite technological advances, lectures remain widely recognised as a vital approach to adult education (Dsouza et al., 2025).

In nursing education, the flipped classroom has proven more effective than traditional lectures in improving students’ learning experience and academic performance, and it fosters students’ competence development (Andargeery et al., 2024; Park & Suh, 2021). It has a positive effect on learning motivation, metacognitive skills, self-directed learning and the development of core competencies (Fan et al., 2020). Students develop a deeper understanding of concepts, increased confidence, improved retention of knowledge and become more reflective on their practices (Busebaia & John, 2020). With the use of a flipped classroom, a positive change in students’ academic performance is reported (Banks & Kay, 2022; Barranquero-Herbosa et al., 2022), improved clinical competence, critical thinking ability (Park & Suh, 2021) and improved communication, collaboration skills, and students made an effort to create a positive classroom atmosphere (Zhang et al., 2025). Banks and Kay (2022) reported on student satisfaction and self-efficacy while following a flipped classroom approach. However, some challenges were reported regarding the use of flipped classrooms in nursing education. It requires more time and much effort from both students and educators, as well as access to technology and a physical classroom for in-class set-up (Kazeminia et al., 2022). From the lecturers’ perspective, the flipped classroom requires careful planning and organisation (Joy et al., 2023). Students participation in pre-class activities may be low, as some experience difficulty completing activities; some struggle to understand the pre-course materials, while others fail to self-regulate (Liu et al., 2024a). Moreover, students with poor foundational knowledge struggled to keep up with others, and the flipped classroom was found to be unsuitable for skills practice in nursing (Zhang et al., 2025).

Human anatomy courses play a foundational role in nursing education, as they give students a comprehensive understanding of the structure and function of the human body (Yao et al., 2024). The flipped classroom is among the methods to consider for teaching the human anatomy curriculum. This could be because it allows for deeper levels of learning according to Bloom's taxonomy (Kazeminia et al., 2022). Furthermore, the flipped classroom approach can compensate for the reduction of anatomy educational hours (El Sadik & Al Abdulmonem, 2021) and enhance students’ confidence to pursue independent study and grasp challenging anatomical concepts (Xiao, 2024). On the other hand, it promotes creativity in anatomy classes and improves peer-assisted learning (Joy et al., 2023). Compared to the didactic lecture format, the flipped classroom approach enhances nursing students’ performance and satisfaction in an anatomy and physiology course (Joseph et al., 2021).

Previous research has predominantly focused on the flipped classroom in general higher education, nursing education and anatomy education for medical students; however, its specific use in anatomy education among nursing students remains understudied. These experiences, perceptions and outcomes may not be directly relevant to studying human anatomy in nursing, as the subject covers a vast amount of content and is more challenging for students due to the complexity of anatomical and physiological terminology and concepts (Satoh et al., 2023). Moreover, there is no evidence from sub-Saharan Africa on the use of flipped classrooms in the anatomical education of nursing students, thereby creating empirical and population gaps. Therefore, this study aims to explore and describe nursing students’ experiences of a flipped classroom in a human anatomy course. This study was conducted among students who had not previously experienced a flipped-classroom practice. It is essential that understanding their experiences aids educators in identifying what works well, how to improve the flipped classroom approach, and what support is necessary for its effective adoption in human anatomy education for nursing students.

## Methods

### Research Design

The study employed an exploratory, descriptive qualitative design, which was appropriate given the limited knowledge of the topic in the research setting; thus, there is a need for deeper exploration. The constructivist paradigm guided the methodological orientation, while the Standards for Reporting Qualitative Research (SRQR) directed the study's reporting (O’Brien et al., 2014).

### Theoretical Framework

The flipped classroom is a learning model that aims to replace the traditional approach, where students are usually passive and depend solely on the transfer of information (Erbil, 2020). Consequently, this study adopts constructivism as its theoretical foundation. Generally, the design of the flipped classroom is based on constructivist learning (Ruzafa-Martínez et al., 2023). In constructivism, nursing educators serve as learning facilitators who encourage collaboration and teamwork while guiding students in acquiring their knowledge (Andargeery et al., 2024). The flipped classroom encourages engagement, cognitive processing, learning orientation, motivational orientation and readiness to learn (Shroff et al., 2021), which illustrates a constructivist approach to learning. In the context of this study, the activities provided to students during flipped classes aimed to promote active learning, one of the pillars of constructivism, by placing students at the centre and encouraging them to reflect on their learning.

### Implementation of the Flipped Classroom in the Human Anatomy Course

In the current study setting, the human anatomy course is compulsory for students in the Bachelor of Nursing Science Clinical Honours programme. The course aims to help students master anatomical knowledge of all body systems and apply it in clinical practice. Students register for human anatomy as a year-long first-year course with four hours of class contact time per week, per semester, primarily taught via traditional lectures, with anatomical models as teaching aids. The flipped classroom approach was used once per week in a second-semester undergraduate nursing human anatomy course, whereas the remaining days followed the traditional approach. The lecturer aligned the flipped classroom approach with active learning and constructivist teaching, providing pre-class, in-class and post-class activities to facilitate engagement, exploration, explanation, elaboration and evaluation (Robertson, 2022). The three phases and activities for a flipped classroom in the human anatomy course are shown inFigure 1.

**Figure 1.**
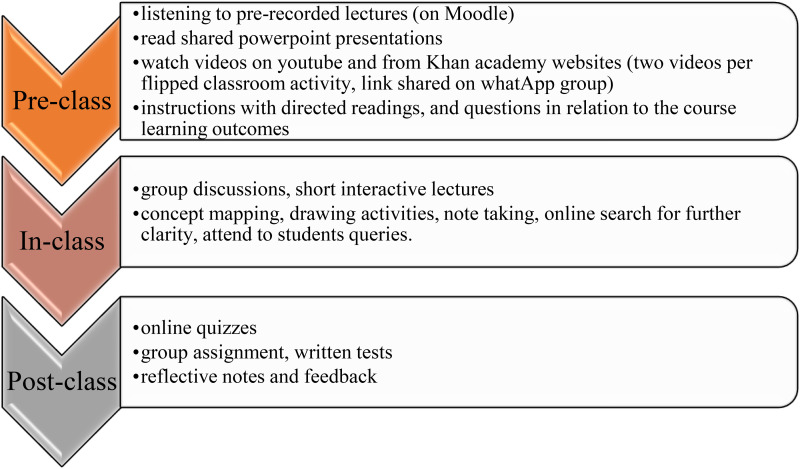
Three phases and their activities for a flipped classroom in the human anatomy course. Source: Authors’ creation.

Research Question: ‘How do nursing students experience using a flipped classroom in a human anatomy course?’

### Participants

The target population of this study comprised 66 second-year nursing students who completed the human anatomy course in 2023 at a public university campus in Namibia. Given that human anatomy is a compulsory foundational course in the Bachelor of Nursing Clinical Honours programme, the sample was obtained through convenience sampling, selecting readily available students (Moser & Korstjens, 2018). Data saturation determined the number of participants, which was reached when no new evidence emerged from the data collection and analysis, thereby providing maximum information on the phenomenon under study (Saunders et al., 2018). Data saturation was reached with the sixteenth participant; however, two additional interviews were conducted to confirm saturation, resulting in a sample of 18 participants.

### Inclusion and Exclusion Criteria

The study included students who had experienced the flipped classroom approach in a human anatomy course. Excluded were second-year nursing students who were unavailable during the data collection period and those who chose not to participate.

### Data Collection

Following ethical clearance and permission from the school ethics committee, the researchers shared information about the study in a WhatsApp group for second-year nursing students. In addition, they directly approached students during their theoretical block on campus. Interested students contacted the researchers and agreed on an interview date and time. Data were collected through semi-structured individual interviews. All researchers conducted interviews in English, with an average duration of 32 min. An interview guide was followed, which had been piloted with two participants to identify major flaws before the main study. The central question in the interview guide was “*Tell me your experiences with the use of a flipped classroom in the human anatomy course”? *During the interviews, prompts were used to facilitate discussion and elicit detailed responses, thereby yielding rich data from participants. Data were collected from April to June 2024 and audio-recorded with participants’ consent, either using a smartphone voice recorder or via recorded voice calls. All researchers are female; the first researcher has a PhD in nursing education, while the second holds a Bachelor of Nursing Science (clinical) (honours). All have undergone qualitative research training and are well-versed in its methods.

### Data Processing and Analysis

The interview audio recordings were transferred to a personal computer and transcribed verbatim to produce a written transcript. The transcription was done verbatim, using a cloud-based Word programme within the Microsoft 365 software suite. Data analysis was conducted manually, following six stages of the iterative nature of analysing interview data (Knott et al., 2022), which led to the formation of themes and their subthemes (Table 1).

**Table 1.** Application of Knott et al. (2022), the Iterative Nature of Analysing Interview Data in the Data Analysis.

Stages	Application
1. Recalling the research question	This initial phase reminded the researchers of what they would be looking for in the data. The research question was ‘How do nursing students experience using a flipped classroom in a human anatomy course?’
2. Immersing	The researchers read through the interview transcripts several times to familiarise themselves with the data.
3. Coding	As researchers read through the transcripts, they developed codes and labels.
4. Interpreting	Researchers delve into developed codes, reflecting on them to answer the question of how these codes help address the research question: What do these codes mean? What participants’ direct quotes will be used to support subthemes to be developed from these codes? How will these codes be unpacked further?
5. Synthesising and memoing	The important aspect at this stage was to draw the data together by examining the similarities and differences in the codes. Researchers noted their reflections on these differences and similarities, then applied synthesis to group the codes into subthemes and themes.
6. Writing	The researchers compiled the final report on their findings by interpreting subthemes and themes, and contextualising them within existing literature.

### Study Rigour

The rigour of the study was established in accordance with the recommendations for establishing rigour in qualitative inquiry (Morse, 2015). This included the following strategies: prolonged engagement, persistent observation, thick and rich description, peer review or debriefing, clarifying researcher bias, member checking and triangulation. Prolonged engagement was achieved by collecting data over three months, which was sufficient time to help build rapport with participants and allow the data to saturate. Persistent observations involved paying attention to details about the phenomenon under study during data collection and using prompts and probes. Thick, rich descriptions were provided through rationales and detailed accounts of methods and choices. Moreover, the SRQR checklist was followed to report findings. Peer review and debriefing were conducted to provide critique, validation and feedback on the proposal, interview guide and draft findings. To clarify researcher bias, the researchers practiced reflexivity by making notes in their research diary and field notes on their reflections regarding the research process, while acknowledging personal biases and preconceptions related to the use of the flipped classroom in the human anatomy course. Member checking was conducted by sharing data and themes with participants to prevent misunderstandings of facts and experiences. Researcher triangulation was employed in this study to minimise biases in data collection, analysis and reporting of findings (Donkoh, 2023). All researchers were involved in the conceptualisation of the study, data collection and analysis, and held regular consensus meetings to agree on choices and decisions made.

### Ethical Consideration

The study received ethical clearance from the school's ethics committee at the campus level and permission to conduct the research. The study adhered to the four ethical principles of Dhai and McQuoid-Mason (2011), namely the principles of respect and autonomy, non-maleficence, beneficence and justice. In addition, the revised Declaration of Helsinki guidelines for medical research that involved human participants were adhered to WMA (2024). Information provided to participants about the study included the benefits and risks of participation, and they signed an informed consent form prior to participating and being audio recorded. Participants were informed of their right to withdraw from the study at any point without further explanation. No names or personal identifying information, such as student numbers or national identification numbers, were recorded; participants were referred to by a numerical code (e.g., P1).

## Results

### Sample Characteristics

The participants ranged in age from 19 to 33 years old. Among the 18 participants, 11 were female, and seven were male. All participants were in their second year of the Bachelor of Nursing Science (clinical) (honours) programme in 2024 and had completed the human anatomy course in 2023. Table 2 presents the full demographic characteristics of the participants.

**Table 2.** Sample Characteristics.

Participants’ code	Age (in years)	Gender	Repeated human anatomy course
**P1**	23	Female	No
**P2**	19	Female	No
**P3**	20	Male	No
**P4**	23	Female	No
**P5**	20	Male	No
**P6**	19	Female	No
**P7**	24	Male	No
**P8**	20	Male	No
**P9**	21	Female	No
**P10**	33	Male	No
**P11**	21	Female	No
**P12**	22	Female	No
**P13**	23	Female	No
**P14**	20	Male	No
**P15**	19	Female	No
**P16**	24	Male	No
**P17**	27	Female	No
**P18**	32	Female	Yes

## Themes

Three themes were identified through data analysis, with 12 subthemes presented in Table 3.

**Table 3.** The Themes and Sub-Themes.

Themes	Sub-themes
The flipped classroom promotes active learning	Encourages self-regulated learning Promotes a deep approach to learning Enhances cognitive processing Fosters student engagement, collaboration and active participation
The flipped classroom is not the preferred method for learning human anatomy	Content overload Insufficient comprehension of the content. Limited understanding of the flipped classroom approach Technical difficulties
Improving the flipped classroom approach	More recorded lectures Feedback Adjust learning activities to accommodate slower students Ensure adequate support for students.

### Theme 1: The Flipped Classroom Promotes Active Learning

This theme describes nursing students’ experiences with the flipped classroom approach, which encourages them to be actively involved in the learning process of the human anatomy course. It is described in four subthemes as follows;

#### Sub-Theme 1.1: Encourages Self-Regulated Learning

Participants report that they were able to engage with the material independently before attending class, which helped them determine when and how to study it. This way, they felt they were in control of their learning without being rushed. Given sufficient time to review the content independently, students can set goals, determine how to study the materials effectively, and complete the required tasks. Being in control allowed students to consult other sources not prescribed by the lecturer, and better understand the content by spending more time on the most challenging topic. Participant mentioned this: ‘It helped me to develop independent learning skills’ (P9). ‘It allows me to get independent learning skills. I do not have to depend on anyone; I am just on my own’ (P4). ‘I was able to come up with my study plan, which includes what I should do and when to do it’ (P16).

#### Sub-Theme 1.2: Promotes a Deep Approach to Learning

Because sufficient time was given for pre-class learning activities, the flipped classroom approach enabled students to conduct extensive research on the topics and watch videos or recorded lectures multiple times. This method helped them generate follow-up questions to discuss with peers or the lecturer during the in-class session. The flipped classroom approach helped students analyse content by comparing different body systems to gain a better understanding.

‘I had more time to study the complex concepts, googled more about their meaning, and wrote down questions I needed answers to from my colleagues who understand the subject better. Sometimes I ask the lecturer, which leads to a deeper understanding’ (P2). ‘Like when we covered the nervous system, I read about the endocrine system just to know how they relate’ (P16).

Participants noted that the flipped classroom helps them arrive well-prepared and gives them an idea of what the lesson will cover. Preparations include linking the current topic with previous knowledge from secondary school and reading more about the body system to focus on for the day. ‘It helps me read thoroughly about the content, as I try by all means to remember what we were taught in biology, then see what I can expand on through studying anatomy. That way, nothing will be too new to me in class’ (P13).

#### Sub-Theme 1.3: Enhances Cognitive Processing

The flipped classroom approach enhances cognitive processing of content as students participate in in-class activities, such as group discussions, problem-solving, debating, and quizzes, and reflect on the content. The participants mentioned this: ‘flipped classroom opened up my mind, and I started thinking critically because every time I prepare at home with the pre-recorded videos and the notes that were sent by the lecturer, I started delving more into detail by coming up with my ideas’ (P7). ‘It granted me the opportunity to shift my thinking from theoretical to practical, which helped me with critical thinking’ (P10). ‘The lecturer likes asking questions in class, which helped me to think deeply and reflect on what I read before’ (P12).

#### Sub-Theme 1.4: Fosters Student Engagement, Collaboration and Active Participation

Participants reported increased engagement with the course materials and class attendance, facilitated by pre-class and in-class activities. The flipped classroom was also reported to promote learning collaboration among students, in which they engage in discussions with others or complete assessment tasks together. Participants mentioned this: ‘It also helped me engage with my fellow students and discuss more about the content before coming to class’ (P1). ‘I like it because it provides an opportunity to work with my classmates instead of sitting at home or in class and doing it all by myself’ (P8).

Furthermore, active participation during pre- and in-class activities was highly valued. This makes the class engaging and helps avoid academic fatigue. Additionally, the flipped classroom promoted open discussions because students had prior exposure to the content being presented in class. Since students worked on study activities together, they formed teams and identified those who did not understand the content. ‘It helped me to actively participate in class because I had gone through the content already’ (P5). ‘I was able to identify fellow students with similar interests and formed our study group, it is going well so far’ (P13).

### Theme 2: The Flipped Classroom Is Not the Preferred Method for Learning Human Anatomy

This theme presents factors identified by students, explaining why the flipped classroom approach may not be suitable for learning human anatomy. Additionally, it explains how these factors adversely impact the understanding of human anatomy.

#### Sub-Theme 2.1: Content Overload

The human anatomy course has extensive content that covers the entire body; therefore, content overload was the most significant challenge for the students, making it less suitable for the flipped classroom approach. Participants further described being given lengthy recorded lectures and numerous presentations to cover within a short timeframe, which makes it difficult to complete alongside their commitments. This was mentioned: ‘The content was extensive, so I had to further adjust my schedule to dedicate more time to anatomy and less to other subjects’ (P7). ‘We covered many systems; some have a lot of content, which means there was also a lot of homework, and class content is still a lot, but time is short’ (P14).

Students tend not to cover all the content shared by the lecturer as pre-class activities, in order to focus on preparing for the assessment. Furthermore, participants indicated they are selective about which tasks to cover, specifically focusing on aspects they consider important. This is what the participants have said: ‘Sometimes I do not get time to read through all the homework, for example, if I have a test for another subject, I have to study for the test because this subject is just too much (P3). ‘There is much content, and I have social activities and other modules to attend to, so I cannot focus solely on anatomy’ (P6).

#### Sub-Theme 2.2: Insufficient Comprehension of the Content

Participants reported that there were times when they did not understand the recorded lectures and other learning materials provided, and they needed help from fellow students. They added that some topics can only be understood when presented by the lecturer first, rather than being assigned as a pre-class activity to be explained later. The lack of understanding of subject content was related to complex concepts and those that are difficult to comprehend or articulate, combined with a large volume of content. This is what the participants mentioned: ‘Sometimes, when I am doing my self-studies, there are times when I am studying or, let me say, listening to pre-recorded materials, and I reach a point where I do not understand a specific topic that I am studying’ (P9). ‘It was difficult for me to understand the whole content or the whole lesson on my own because there are some words that need to be explained by the lecturer, no matter how much I read, no matter how much I try, I will not understand unless the lecturer intervenes’ (P4). ‘It was difficult to follow a session on the nervous system; the nerves and neurons were not easy to understand in a recorded lecture that we had to listen to’ (P16).

#### Sub-Theme 2.3: Limited Understanding of the Flipped Classroom Approach

Since the flipped classroom was a relatively new teaching approach for the students, they have been struggling to understand how to implement and adapt to it. Their concern was that they were in their first year and had just come from secondary schools where teaching was done using traditional methods, so it was difficult to understand how it worked. This was mentioned*:* ‘I remember the first few weeks when we started with this strategy; it was confusing since it was new to me. I did not know how I could study topics independently first and then teach later’ (P7). ‘Although this new strategy was explained, I was still getting used to attending classes online, watching pre-recorded lectures, or completing online activities, let me say the problem was how to do it’ (P2).

#### Sub-Theme 2.4: Technical Difficulties

Participants reported that attending to the pre-class activities required access to a smartphone or personal laptop and the internet. This was necessary to access the recorded lectures or participate in the online discussions. Those with the correct device and internet access were unsure how to use Moodle to access prerecorded materials, notes, or online activities, as they were accustomed to the traditional teaching method. This was mentioned: ‘Sometimes I had no access to the internet to go through the content’ (P1). ‘As a first-year student, I did not have technological skills. Going on Moodle to search for the prerecorded materials was difficult, and I sometimes encountered network problems’ (P10). ‘It was not possible to do all activities, especially when I found myself in a place with a poor network, and sometimes my data ran out’ (P11).

### Theme 3: Improving the Flipped Classroom Approach

After describing their experiences with the flipped classroom approach in the human anatomy course, participants were invited to suggest improvements; their responses were categorised into four subthemes.

#### Sub-Theme 3.1: More Recorded Lectures

Participants preferred that more recorded lectures be uploaded to the Moodle platform than materials for reading or searching the internet. This will help students access them at their convenience and listen to them multiple times. This was mentioned: ‘I think more recordings by the lecturer explaining the topics should be put on Moodle to allow students to listen over and over’ (P11) maybe share more recordings to use during spare time and also to record more videos to help students with a better understanding’ (P3).

#### Sub-Theme 3.2: Feedback

Participants suggested constructive feedback be given before moving to the next topic, as this will improve the quality of learning in the flipped classroom. Additionally, conversation during the feedback session may help the lecturer determine who understands the content better and who still requires more support. Participants had mentioned: ‘The lecturers should provide more detailed and specific feedback for each topic, including information on how we can improve our learning. Also, I feel they should assess students’ understanding to ensure they've comprehended the content before moving on to the next topic’ (P10).

In addition to feedback from the lecturer to the students, the participants opined that students should be given the opportunity to provide feedback to the lecturer and offer suggestions on how the flipped classroom for each body system can be conducted or improved, given conceptual differences. Participant Five mentioned this: ‘I believe the lecturer should seek feedback from students to identify areas for improvement in the flipped classroom approach. Some methods used in the flipped classroom may not be suitable for studying all body systems. If we have a say, we could suggest what might work better. You know, some systems are easier to understand, like the respiratory system, but for others, they are complicated’ (P5).

#### Sub-Theme 3.3: Adjust Learning Activities to Accommodate Slower Students

Due to differences among students, participants felt they needed varying timeframes to complete the learning activities. Therefore, the activities and the time allocated should be planned with consideration for slower learners. This was mentioned: ‘It is important for lecturers to pace homework according to the slowest students in the class because some adapt quickly while others take more time’ (P7).

Furthermore, participants believed that slow learners should not be left behind: ‘I’m encouraging lecturers to identify slow learners and ensure they understand the content before progressing to the next topic, so all students are on the same page’ (P6).

#### Sub-Theme 3.4: Ensure Adequate Support for Students

Participants felt that support is essential in shaping the learning process during the flipped classroom approach. The academic support students require includes study tips, guidance on searching for information online and the provision of necessary learning materials. This was mentioned: ‘The lecturers should provide support to help students develop self-study skills and self-reflection. They should offer resources such as study guides, checklists, and self-assignment tools to help students go through the pre-class materials independently’ (P2). ‘Students should be provided with previous question papers to review in their free time, and be allocated more time to prepare for tests’ (P8).

## Discussion

The flipped classroom, as an innovative approach in health professions education, yields significant improvements in student learning, particularly in procedural skills and higher-order thinking (Phillips & Wiesbauer, 2022). Using constructivism as its theoretical framework, the current study contributes to this important subject by exploring nursing students’ experiences with the flipped classroom approach in the human anatomy course. The main themes revealed are: the flipped classroom promotes active learning, the flipped classroom is not the preferred method for learning human anatomy, and improving the flipped classroom approach.

Nursing students’ descriptions of their experiences with the flipped classroom indicated that it promotes active learning by encouraging self-regulated learning. These align with findings of Fan et al. (2020) as well as Busebaia and John (2020) on the promotion of self-regulated learning. In the current study, the flipped classroom included pre-class activities that required students to complete tasks independently, such as listening to prerecorded lectures, watching videos, reading, and searching online resources and textbooks. This approach encouraged students to adopt a deep learning approach to human anatomy. The findings are consistent with those of Al Suliman et al. (2025), who reported that students were satisfied with activities such as reviewing handouts and note-taking, as well as watching recorded videos, all of which promoted self-directed learning and significantly enhanced their educational experience. Liu et al. (2024b) reported that the flipped classroom in a physiology class promoted a deep learning motive and strategy, which characterises a deep learning approach. Similarly, the current study revealed that the flipped classroom enhances cognitive processing, fosters student engagement, collaboration, and active participation. This implies that the approach facilitated engagement through activities conducted before, during, and after class. The students appreciated having more time to interact with learning materials, which allows for reflection on the content and the option to form learning groups with classmates while completing pre-class activities. The in-class sessions were also described as interactive, providing opportunities for questions and answers. Furthermore, the current study revealed that the flipped classroom is not the preferred method for learning human anatomy for various reasons identified in the findings. One reason is content overload, in terms of the number of self-study activities and lengthy recorded lectures, which makes it difficult for students to complete all their assigned tasks. Correspondingly, high workload associated with the flipped classroom and the time-consuming nature of completing all activities have been reported in previous research (Joy et al., 2023; Morini et al., 2024). In addition, nursing students in the current study found it difficult to sufficiently comprehend the content due to complex concepts and those that are hard to understand or articulate, combined with a large volume of material. As a solution, they sought help from peers for a simplified explanation and noted down their questions to discuss with the lecturer during the class session. The limited understanding of the flipped classroom approach is another reason why it was not preferred for learning about human anatomy. The flipped classroom approach was not applied to other courses, and students encountered it for the first time in this course. Therefore, a comprehensive explanation of what was expected was provided, along with regular reminders throughout the semester. Similarly, Morini et al. (2024) reported that students had limited understanding of what a flipped classroom involved and were unprepared for the new learning approach. The students’ understanding of the flipped classroom approach may be enhanced by providing a more detailed syllabus that explains why the flipped classroom approach is necessary. Additionally, explain what they could accomplish by adopting flipped classroom activities to improve students’ course experience and encourage engagement. While the flipped classroom approach may be used for intentional content in anatomy (Nurunnabi et al., 2022), in the current study, it was applied simply by considering the sequence in which content is supposed to be delivered to students. No specific topics were deliberately designated for the flipped classroom approach.

In anatomical education, the flipped classroom approach, integrating multimodal digital resources, could help improve students’ long-term learning outcomes and cognitive skills (Xiao & Adnan, 2022). However, in the current study, students experienced technical difficulties, including unfamiliarity with the Moodle platform, where learning materials are shared and a lack of internet connectivity, which prevented them from fully benefiting from digital resources. Therefore, the current findings did not correspond with previous research in this area.

Therefore, improvements are necessary for the successful implementation of the flipped classroom approach in the human anatomy course for nursing students. Students have suggested that the lecturer share more recorded lectures on Moodle rather than assigning tasks that require them to read information from textbooks and other materials. The recorded lectures are more detailed and provide students with the opportunity to listen repeatedly as well as at their own pace. Another aspect for improvement is feedback. In the current study, feedback was incorporated as a post-class activity; however, this was not sufficient for students, who prefer more constructive feedback from the lecturer and also for the lecturer to seek feedback from them. Providing feedback to students is vital for keeping engagement in the flipped classroom, as it offers personalised support and recognises each student's achievements (Masruddin et al., 2024). Feedback is an important aspect of the collaboration process as it facilitates interaction, offers suggestions, and directs students’ thinking during the flipped classroom (Chen et al., 2023). Therefore, provision should be made for feedback on pre-class, in-class and post-class activities. Another suggestion for improving the flipped classroom in the current study was to adjust learning activities to accommodate slower students, with students emphasising that slow students should not be left behind. This suggestion is viable, as a flipped classroom may provide an opportunity for remediation in nursing education (Wilson & Hobbs, 2023). While students in the current study are concerned about slower-performing individuals, a previous study reported that students with higher prior academic achievement experienced worse learning outcomes in the flipped classroom, characterised by lower learning satisfaction (Chen et al., 2023). The current study emphasises the importance of providing sufficient support to students when using the flipped classroom approach. The support requested was more focused on academic issues, such as study tips, guidance on online searches and the provision of learning materials. As preparation for the flipped classroom, students were provided with instructions on how to conduct activities and where to find learning materials. Interestingly, the use of quizzes as a form of support for students during in-class and post-class activities is beneficial as they aid effective learning by providing opportunities for self-evaluation and reinforcing their knowledge (El Sadik & Al Abdulmonem, 2021).

The current study presented unique findings collected in an undergraduate nursing context, in a sub-Saharan African setting that is also resource-constrained. The flipped classroom described was implemented with limited multimedia resources and anatomical models, which differs from what is found in the literature, thereby making these findings unique to nursing education, specifically in bioscience teaching and learning.

### Strengths and Limitations

The study used a qualitative, exploratory and descriptive design, which allowed the researcher to delve deeper into students’ experiences with the flipped classroom; this is therefore regarded as a strength of the study. However, the study employed a convenience sampling technique, which consequently included only students who had access to WhatsApp during the data collection period and those who attended classes on days the researcher visited to introduce the research project. These considerations should be taken into account when interpreting the current findings and evaluating their transferability.

### Implications for Practice

The findings of this study are unique in that they contribute to the existing evidence on the use of flipped classrooms in higher education, nursing and medical training, with a specific focus on learning human anatomy among nursing students. Henceforth, the findings have implications for international, national and local contexts, providing evidence for modifications to pre-class, in-class and post-class activities. Based on this study's findings, students are encouraged to adopt the flipped classroom approach, as it promotes active learning and is expected to improve learning outcomes in human anatomy. Furthermore, students should organise their activities effectively to ensure they attend to pre-class tasks and consult peers, lecturers and support staff when facing challenges. Findings provided evidence that nurse educators need to provide constructive feedback on all activities assigned to students in the flipped classroom to maximise its effectiveness. Another implication for practice is that the flipped classroom should be integrated into a blended learning approach alongside other active learning methods. The findings of the current study may inform the development of a step-by-step guide for nurse educators planning to implement the flipped classroom approach in human anatomy instruction.

## Conclusions

It is concluded that nursing students have both positive and negative experiences with the flipped classroom approach in human anatomy. On the positive experience, the flipped classroom promotes active learning by promoting self-regulation, a deep approach to learning and cognitive processing of content, while fostering student engagement, collaboration and active participation. However, students did not prefer learning human anatomy through the flipped classroom approach because of content overload, limited understanding of the approach, insufficient comprehension of the material and technical difficulties. Moreover, the findings indicate that the flipped classroom approach in human anatomy requires improvement by offering more recorded lectures, providing students with feedback and allowing lecturers to solicit feedback. Additionally, it is advisable to consider the needs of slow students when planning learning activities and to ensure sufficient support is provided. Future researchers might explore how the flipped classroom can be utilised to enhance the application of human anatomy in clinical practice among nursing students.
